# Cysteine, Glutathione, and Thiol Redox Balance in Astrocytes

**DOI:** 10.3390/antiox6030062

**Published:** 2017-08-03

**Authors:** Gethin J. McBean

**Affiliations:** School of Biomolecular and Biomedical Science, Conway Institute, University College Dublin, Dublin, Ireland; gethin.mcbean@ucd.ie; Tel.: +353-1-716-6770

**Keywords:** cysteine, cystine, cysteamine, cystathionine, glutathione, *x_c_*^−^ cystine-glutamate exchanger, transsulfuration

## Abstract

This review discusses the current understanding of cysteine and glutathione redox balance in astrocytes. Particular emphasis is placed on the impact of oxidative stress and astrocyte activation on pathways that provide cysteine as a precursor for glutathione. The effect of the disruption of thiol-containing amino acid metabolism on the antioxidant capacity of astrocytes is also discussed.

## 1. Introduction

Thiol groups, whether contained within small molecules, peptides, or proteins, are highly reactive and prone to spontaneous oxidation. Free cysteine readily oxidises to its corresponding disulfide, cystine, that together form the cysteine/cystine redox couple. Similarly, the tripeptide glutathione (γ-glutamyl-cysteinyl-glycine) exists in both reduced (GSH) and oxidised (glutathione disulfide; GSSG) forms, depending on the oxidation state of the sulfur atom on the cysteine residue. In the case of proteins, the free sulfhydryl group on cysteines can adopt a number of oxidation states, ranging from disulfides (–S–S–) and sulfenic acids (–SOOH), which are reversible, to the more oxidised sulfinic (–SOO_2_H) and sulfonic acids (–SOO_3_H), which are not. These latter species may arise as a result of chronic and/or severe oxidative stress, and generally indicate a loss of function of irreversibly oxidised proteins. Methionine residues oxidise to the corresponding sulfoxide, which can be rescued enzymatically by methionine sulfoxide reductase [1]. Again, excessive oxidation produces methionine sulfone that cannot revert to the reduced state.

**Figure 1:**
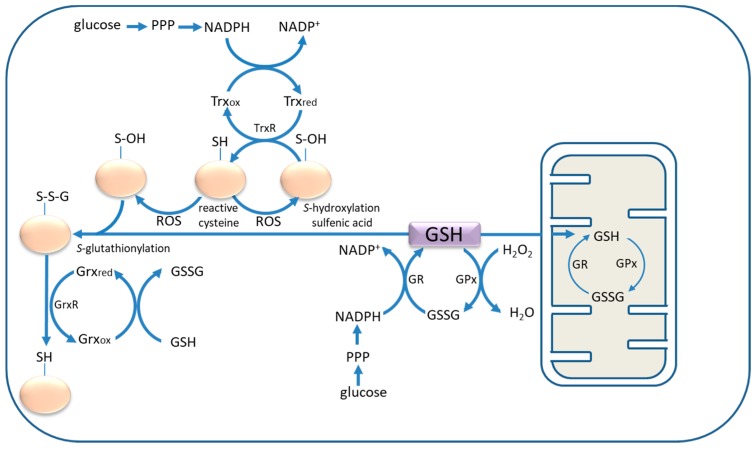
The redox cascade involved in the protection of protein sulfhydryl groups from oxidation. Thioredoxin reductase (TrxR) catalyses the transfer of electrons from reduced thioredoxin (Trx_red_), which is regenerated by transfer of electrons from NADPH to oxidised thioredoxin (Trx_ox_). Alternatively, proteins may become glutathionylated (Pr-SSG), in which case glutaredoxin reductase (GrxR) catalyses the transfer of electrons from reduced glutaredoxin (Grx_red_), which is, in turn, regenerated by oxidation of reduced glutathione (GSH) to glutathione disulfide (GSSG). GSH also participates in the glutathione redox cycle, involving glutathione peroxidase (GPx) and glutathione reductase (GR), in a reaction coupled to oxidation of NADPH that is supplied by the pentose phosphate pathway (PPP). The GSH redox cycle takes place in the cytosol and in mitochondria. ROS, reactive oxygen species.

The protection of protein sulfhydryl groups from oxidation relies on the antioxidant capacity of a network of redox couples. Paramount amongst these are GSH/GSSG and reduced and oxidised thioredoxin (Trx) that operate in tandem with enzymes, including glutathione reductase (GR), glutathione peroxidase (GPx), glutaredoxins (Grx), thioredoxin reductases (TrxR), and peroxiredoxins (Prx) in order to maintain redox balance. Electrons are passed between the components of a redox cascade, in which the ultimate electron donor is NADPH (Figure 1). Neurons and astrocytes oxidise glucose via the pentose phosphate pathway in order to maintain adequate levels of NADPH for antioxidant activity [2,3]. The ratio of GSH to GSSG is normally 90%, but the balance shifts in favour of GSSG during ageing and as a consequence of prolonged oxidative stress, in which case it may be as low as 50% [4].

Characterising the relationship between the cysteine/cystine and GSH/GSSG redox couples is a prerequisite to a full understanding of antioxidant defence in astrocytes. In the sections that follow, current evidence on the sources and supply of cysteine for GSH in astrocytes under normal and activated conditions is discussed. However, whilst this review is primarily about the relationship between cysteine and GSH in astrocytes, Trx deserves mention because of its close association with GSH in astrocyte antioxidant defence. The main isoform of the protein is Trx1; a general account of its action in thiol redox homeostasis is provided in a recent review [5]. The selenoenzyme, thioredoxin reductase (TrxR), returns Trx to its reduced (active) state (Figure 1), and the gene coding for this enzyme is a recognised target of the antioxidant response transcription factor, nuclear factor (erythroid-derived 2)-like 2 (Nrf2) [6]. In a comparison of TrxR activity in neurons and glia, it is reported that during *tert*-butyl hydroperoxide-induced oxidative stress, the activity and protein content of TrxR was increased in cortical astrocytes in culture, but not in neurons [7]. In terms of astrocyte protection from oxidative stress, it has recently been shown that Trx1 supports the antioxidant capacity of the 2-cysteine peroxiredoxins by maintaining their expression [8].

## 2. Cysteine and Glutathione: In Situ Antioxidants and Neuronal Protection

Cysteine is the immediate precursor of GSH, hydrogen sulfide, and taurine, each of which have significant antioxidant and/or neuroprotective properties [9,10,11]. Within cells, the amino acid exists primarily in the reduced form, cysteine, due to the comparatively higher redox potential (i.e., “reducing power”) of the GSH/GSSG redox couple [1]. The intracellular concentration of free cysteine is in the low micromolar range, which is an order of magnitude lower than that of GSH. Extracellularly, the more oxidising conditions favour a predominance of cystine over cysteine, where at a typical concentration of 40–50 µM, it is more plentiful than either GSH (2.8 µM) or GSSG (0.14 µM) [1]. The extracellular cysteine/cystine ratio becomes more oxidised during ageing, which is believed to heighten the susceptibility to disease [12].

**Figure 2:**
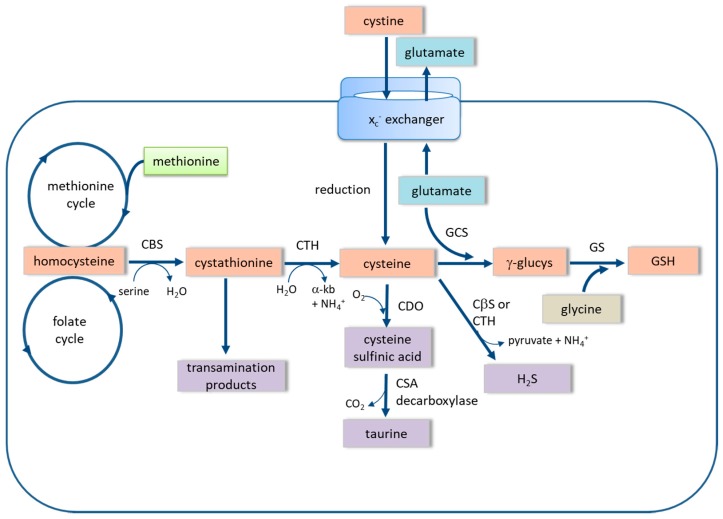
The pathways of synthesis and metabolism of intracellular cysteine. Cystine is imported in exchange for glutamate via the *x_c_^−^* cystine-glutamate exchanger and is reduced to cysteine. Cysteine is also generated from methionine as a product of the trans-sulfuration pathway by action of cystathionine-β-synthase (CβS) and cystathionine-γ-lyase (CTH), respectively. Homocysteine and cystathionine are intermediates of the trans-sulfuration pathway. Glutathione (GSH) is synthesised from cysteine and glutamate, by action of γ-glutamylcysteine synthase (GCS), forming γ-glutamylcysteine (γ-glucys), followed by the addition of glycine in a reaction catalysed by glutathione synthase (GS). Oxidation of cysteine to cysteine sulfinic acid (CSA) is catalysed by cysteine dioxygenase (CDO), followed by decarboxylation to taurine. Hydrogen sulfide (H_2_S) is formed from cysteine by either CβS or CTH. α-kb, α-ketobutyrate.

Astrocytes contain one of the highest cytosolic concentrations of GSH (8–10 mM in primary astrocytes) amongst mammalian cells [13,14,15]. Here, GSH performs the dual role of antioxidant protection in situ and the maintenance of the antioxidant capacity of neurons. Considerable effort has gone into establishing the inter-relationship between astrocytic and neuronal GSH levels. The two GSH biosynthetic enzymes, γ-glutamate cysteine ligase (also known as γ-glutamylcysteine synthase) and glutathione synthase, are highly expressed in astrocytes, and together generate GSH from its immediate amino acid precursors, glutamate, cysteine, and glycine (Figure 2). Through an extensive series of metabolite labelling studies, Dringen and colleagues [9,16,17] have traced the export of GSH from astrocytes via the multidrug resistance protein 1, followed by the separation of the γ-glutamyl group from cysteinylglycine by action of the membrane-bound enzyme, γ-glutamyltransferase. Cysteinylglycine is broken down into cysteine and glycine by action of an ectopeptidase, which are then taken up by neurons. The γ-glutamyl group is recycled into astrocytes coupled to an acceptor amino acid, and ultimately re-generates glutamate via oxoproline. This series of reactions completes the γ-glutamyl cycle, as first elucidated by Alton Meister and colleagues during the 1960s [18]. The net result of this seemingly tortuous process is that astrocytes provide neurons with the components to generate GSH in situ, as neuronal glutamate is also derived from astrocytes in the form of glutamine [19].

The regulation of GSH synthesis occurs at the level of γ-glutamate cysteine ligase (GCL). This enzyme is composed of catalytic (GCLc) and modifier (GCLm) subunits that are coupled by a disulfide bond that is important in regulating substrate and inhibitor interactions with the protein [14]. The rate of de novo synthesis of GSH from its amino acid precursors depends upon the activity of the enzyme, its level of expression within the cell, the availability of substrates, and feedback inhibition by GSH [20]. As the intracellular concentration of cysteine is typically much lower than that of glutamate or glycine, cysteine is considered the rate-limiting precursor for GSH synthesis. It follows that the GSH-mediated antioxidant capacity of both astrocytes and neurons hinges on the availability of cysteine to generate GSH in astrocytes.

## 3. The *x_c_^−^* Cystine-Glutamate Exchanger

In early work, it was established that cultured astrocytes take up cystine in preference to cysteine, in correspondence with the fact that extracellular oxidising conditions favour a predominance of cystine over cysteine [9]. Mechanistically, cystine is imported in exchange for glutamate by a plasma-membrane transporter known as the *x_c_^−^* cystine-glutamate exchanger, and is reduced intracellularly to cysteine. In vitro experiments employing primary astrocytes confirmed that the exchanger was active in these cells, and that uptake of cystine was rate-limiting for the synthesis of GSH [21,22]. However, with the emergence of xCT (the functional subunit of the *x_c_^−^*-exchanger) knock-out animals, the close association between the exchanger and GSH levels has been questioned. xCT^−/−^ mice are healthy, but display a higher plasma cystine and lower plasma GSH concentration than normal mice [23]. Importantly, an absence of the exchanger does not lead to a reduction in GSH in the hippocampus, nor is there evidence of oxidative stress, as would be expected if the *x_c_^−^*-exchanger was critical for supplying cysteine for GSH [24]. On the other hand, the overexpression of xCT in astrocytes increases both the synthesis and release of GSH that protects neurons against oxidative damage in co-culture experiments [25]. Furthermore, astrocytes derived from sut/sut mice (an xCT loss-of-function mutant) display increased oxidative stress and reduced rates of proliferation [25]. The failure to thrive could be rescued by adding the thiol donor, β-mercaptoethanol, indicating that the cells suffered from thiol deficiency. In attempting to rationalise the discrepancy between the various reports, it would appear that astrocytes in isolation are dependent upon the *x_c_^−^*-exchanger for cysteine to generate GSH, whereas, in vivo, or in mixed cultures, the relationship is less critical. As discussed by Conrad and Sato [26], the maintenance of an intracellular/extracellular cysteine/cystine redox balance may be more significant in vivo than channeling cysteine into GSH [27]. One can conclude, therefore, that the ability of animals to survive in the absence of a functional *x_c_^−^*-exchanger suggests that alternative pathways or compensatory mechanisms operate to maintain an adequate supply of intracellular cysteine and GSH.

A significant component of the *x_c_^−^*-exchanger is the release of glutamate that occurs concurrently with the inward passage of a molecule of cystine. This feature places the exchanger at the interface between redox regulation and possible neuronal damage via the hyper-activation of glutamate receptors, unless the glutamate is re-cycled efficiently via high affinity glutamate transporters. Indeed, xCT deletion has a significant impact on the release of glutamate that affects cerebral homeostasis and well-being. For example, the extracellular concentration of glutamate in the hippocampus is significantly decreased in *xCT*^−/−^ mice [24]. In addition, the pharmacological blockade of the *x_c_^−^*-exchanger decreases extracellular glutamate, whereas in vivo dialysis of cysteine precursors increases the level of extracellular glutamate [24]. Glutamate released via the exchanger is important physiologically in terms of the activation of extra-synaptic metabotropic and N-methyl-D-aspartate (NMDA) glutamate receptors [28,29], but can have pathological consequences, as has been reported in the case of microglia and macrophages [30] and glioma cells, which are discussed below.

The first observation that the expression and functional activity of the *x_c_^−^*-exchanger increased in response to oxidative stress was reported in macrophages [31], which was later confirmed to be the case in retinal Müller glial cells [32]. Efforts have been made to link changes in expression of the *x_c_^−^*-exchanger and GSH synthesising enzymes during oxidative stress with the activation of Nrf2, but with differing results. Nrf2 is more highly expressed in astrocytes than in neurons. For instance, cortical neurons express 100–1000-fold less Nrf2 than astrocytes [33]. Accordingly, the vulnerability of astrocytes to oxidative stress increases in Nrf2 deficient animals, whereas neurons are not affected [34]. Shih et al [35] reported that in mixed glial cultures, the overexpression of Nrf2 results in the upregulation of xCT and enzymes involved in GSH synthesis (GCL and glutamine synthase) that leads to the protection of neurons from oxidative stress. Lewerenz et al. [36] provide evidence that the antibiotic, ceftriaxone, increases the *x_c_^−^*-exchanger and GSH in rat cortical and spinal astrocytes via the activation of Nrf2. However, a more recent study, in which quantitative proteomics was coupled with stable isotope labeling of amino acids in mouse primary astrocytes during oxidative stress, revealed that neither the *x_c_^−^*-exchanger, nor enzymes of GSH synthesis, were included in a list of 29 differentially regulated proteins [37]. The conclusion was drawn that catalase, prostaglandin reductase-1, and peroxiredoxin-6 were the principal targets of Nrf2-mediated antioxidant protection in astrocytes. Nonetheless, since it is known that GCL is a target of Nrf2 in astrocytes [38], it is worth speculating that the Nrf2-mediated stimulation of GSH/xCT in astrocytes may be geared more towards maintaining thiol redox homeostasis of the external environment (and neuroprotection), rather than intracellular antioxidant defence. xCT is also regulated by protein kinase A, as it is reported that the depletion of GSH in the presence of dibutyrylcAMP increases the activity of the exchanger by sevenfold in primary astrocyte cultures [39].

*The x_c_^−^-exchanger in gliomas and astrocytomas.* During the 1990s, interest in the function of the *x_c_^−^*-exchanger grew rapidly following reports that glioma cells display an enhanced activity of the exchanger [40], and that inhibition of the exchanger with sulfasalazine both depleted GSH levels and slowed tumor growth in vivo [41]. Thus, conclusions were drawn that the primary function of the exchanger in the context of tumor cells was to deliver intracellular cysteine to furnish additional supplies of GSH. Similar evidence linking exchanger activity with GSH supply came from comparative investigations on tumors in other organs [42,43]. One of the curiosities regarding the *x_c_^−^*-exchanger in gliomas is that the exchanger is upregulated, yet high affinity glutamate transporters are either absent or mislocalised. Thus, the *x_c_^−^*-mediated release of glutamate brings a pathological exposure of neurons to the excitotoxic effects of an increase in extracellular glutamate [40,44]. In theory, the inhibition or knock-down of the exchanger should confer the dual benefits of a blockade of glutamate release and a limitation in provision of cysteine for GSH, which should prove effective in the treatment of, for instance, brain tumours. Indeed, considerable effort has gone into the search for therapeutic strategies to prevent glutamate-mediated toxicity in brain tumours. In 2008, Savaskan and colleagues [45] reported that the siRNA-mediated knock-down of xCT in gliomas prevented oedema and curtailed neurodegeneration. Others have used a more pharmacological approach to target the exchanger. Foremost amongst these is sulfasalazine, which is effective at inhibiting the *x_c_^−^*-exchanger in cell experiments and animal models [44]. Unfortunately, it has proven unsuitable for clinical applications and was withdrawn from a phase 1/2 prospective randomised study in 2008 [46]. A more promising molecule that has emerged in recent years is erastin, which is a potent and selective inhibitor of the *x_c_^−^*-exchanger [47]. Interestingly, erastin also blocks the regulatory enzyme of the trans-sulfuration pathway, an alternative mechanism for the provision of intracellular cysteine (see below). The upshot is that erastin produces a greater decrease in GSH than could be produced by the blockade of the exchanger alone [48]. Accordingly, the sensitivity of glioma cells to the anti-cancer drug, temozolomide, was potentiated when co-applied with erastin [48].

## 4. The Trans-Sulfuration Pathway

It is increasingly recognised that methionine is a significant source of cysteine for GSH in astrocytes [49,50,51]. The conversion of methionine to cysteine, known as the trans-sulfuration (TS) pathway, occurs via homocysteine and cystathionine as intermediates (Figure 2). Current estimates are that TS supplies approximately one third of the cysteine required for GSH under normal conditions in both primary astrocytes and glioma cells. Moreover, flux through the pathway is increased in response to oxidative stress, or when the *x_c_^−^*-exchanger is blocked, meaning that, under these conditions, a greater percentage of cysteine for GSH originates from methionine rather than the extracellular compartment [50,51]. These observations have prompted the view that the TS pathway acts in a reserve capacity in astrocytes to boost GSH production when needed. Interest in the TS pathway and the *x_c_^−^*-exchanger in the context of the regulation of GSH levels has revealed that the association between the two processes may be tissue-specific. For example, Kang et al. [52], report that, in hepatocytes, the inter-relationship between these two providers of cysteine seems to be the reverse of that observed in astrocytes. At low levels of GSH, the TS pathway is the main supplier of cysteine, but, should demand increase, the expression of the *x_c_^−^*-exchanger goes up. The compensatory mechanism implies that there must be a signalling interaction between these two suppliers of cysteine for GSH that warrants further investigation.

The key components of the TS pathway are two pyridoxal phosphate-dependent enzymes, cystathionine-β-synthase (CBS) and cystathionine-γ-lyase (also known as cystathionase; CTH) that catalyse the conversion of homocysteine and serine to cysteine and α-ketobutyrate via the intermediate cystathionine (Figure 2). In the brain, CTH is the principal regulator of the pathway, whereas CBS fulfils this role in peripheral tissues. This view stems from the observation that CTH is less widely expressed in brain than CBS, and therefore the expression and activity of CTH is rate-limiting in respect of flux through the pathway. Evidence in support of CTH’s regulatory role comes from the fact that the intracellular concentration of cystathionine is higher in brain than in other organs [49,53]. CBS and CTH, both being pyridoxal phosphate-dependent enzymes, have a broad spectrum of substrate specificities. In fact, either enzyme can generate hydrogen sulfide (H_2_S) using cysteine or homocysteine as substrate [54]. It is believed that, in astrocytes, CBS is more important than CTH in supplying H_2_S, whereas in vascular tissues CTH fulfils this purpose [53]. However, more recent analyses of H_2_S production in human astrocytoma cells conclude that a third H_2_S-producing enzyme, 3-mercaptopyruvate transsulfurase, generates the bulk of the gas physiologically [55].

CBS is distributed ubiquitously in adult brain, but with greatest expression in the molecular layer of the cerebellum and in the dentate gyrus [56]. During early development, the enzyme is expressed in neuroepithelial cells, but at late embryonic and juvenile stages, it is primarily expressed in astrocytes. Miyamoto et al. [57] noted that the expression of CBS was low in astrocytes in vitro, but that this increased significantly in neuron-astrocyte co-cultures. The production of H_2_S also increased, but no details were provided on whether the GSH levels were similarly affected.

An inherited deficiency in CBS results in a pathological accumulation of homocysteine in tissues and organs that is known as homocystinuria. In the CNS, the condition is characterised by mental retardation, the incidence of seizures, and psychiatric disturbances. An increase in homocysteine in the brain would be expected to cause oxidative stress due to a limited supply of cysteine for GSH. This has been confirmed in a recent study by [58]. Following a 24 h incubation period of primary astrocytes with homocysteine, the cells showed morphological changes, consistent with activation, that were accompanied by a significant decrease in GSH content and a reduction in the activities of antioxidant enzymes, superoxide dismutase, glutathione peroxidase, and hemeoxygenase-1. It was concluded that the observed increase in homocysteine in the cerebrospinal fluid of Alzheimer’s disease and Parkinson’s disease patients may be a cause of astrocyte activation and a consequent impairment in thiol-based antioxidant capabilities [59]. The oxidation of accumulated homocysteine may also contribute to oxidative stress. Although homocysteine oxidises at a slower rate than cysteine, this is accelerated by low concentrations of cysteine or cystine [60], and would likely exacerbate the disruption to redox homeostasis caused by GSH depletion.

The function of cystathionine in the brain may be broader than merely serving as an intermediate in the TS pathway. In their investigation of precursors for GSH synthesis in astrocytes, Kranich and colleagues [9] reported that cystathionine could partially replace cystine in the medium, implying that cystathionine was actively accumulated by the cells. More recently, cystathionine has been identified as a substrate for the *x_c_^−^*-exchanger, and, indeed, interesting questions are emerging on the role of this amino acid in the regulation of thiol balance in the brain [61]. One of the conundrums surrounding cystathionine is whether it serves as a direct precursor of cysteine for GSH, or whether it may exchange for incoming cystine at the level of the *x_c_^−^*-exchanger. Indeed, it is also uncertain whether cytosolic cystathionine itself is derived primarily from methionine by the TS pathway, or if it enters in place of cystine via the exchanger. In cells that are naturally deficient in TS enzymes (for example, the thymus), cystathionine is imported directly, as confirmed by the observation that the cytosolic concentration of the amino acid in thymus is severely depleted following xCT knock-out [61]. Curiously, this study revealed that the concentration of cystathionine in the cerebellum increased following xCT knock-out, which may be due to the upregulation of the TS pathway, as has been observed in the case of the pharmacological blockade of the exchanger [50]. Another plausible explanation is that the export of cystathionine as the counter-species via the exchanger is blocked in xCT^−/−^ cells. Either way, the results from the investigations to date suggest that the supply demand of cystathionine may be cell type-specific, and much remains to be discerned in regard to its function in the brain. Moreover, several biologically-active cyclic transamination products of cystathionine have been identified that may, in time, emerge as regulators of oxidative or immune function in astrocytes [50].

*H_2_S in astrocytes.* H_2_S has several physiological and pathological actions in the brain, which include an ability to protect neurons from oxidative stress and a glutamate-mediated excitotoxicity [62,63]. In terms of antioxidant activity, H_2_S is known to increase GSH formation by sulfhydration or the sulfation of cysteine residues in GSH-associated enzymes [62]. Estimates of the relative capacity for H_2_S production amongst brain cells in vitro indicate that the bulk of H_2_S is generated by CBS in astrocytes [53], and that the gas is released from astrocytes in response to neuronal activation [63]. Amongst the pleiotropic actions of H_2_S that have been recorded to date is the dilation of cerebral vessels, an elevation of intracellular calcium concentration in neurons and glia, the facilitation of long term potentiation, and synaptic remodelling [63,64]. It is evident that the manipulation of H_2_S may have considerable potential as a therapeutic target that will become clearer once there is a better understanding of the factors that control its synthesis and the relationship between H_2_S production and maintenance of GSH in astrocytes.

## 5. Astrocyte Activation and Thiol Antioxidants

Astrocyte activation, also known as ‘reactive gliosis’, is a significant component of the brain’s immune response. In general, astrocyte activation takes place over a slower timescale than that of microglial cell activation, and frequently occurs in response to the release of pro-inflammatory cytokines and immune mediators from activated microglial cells. Significant changes occur as a result of astrocyte activation that likely compromise the thiol antioxidant capacity of the cells. For example, an investigation into the responsiveness of primary astrocytes to stimulation by lipopolysaccharides (LPS) showed that the rate of glucose oxidation through the pentose phosphate pathway increased by more than sixfold, leading to an elevation in the production of NADPH [2]. It was further demonstrated that the increase in NADPH prevented the oxidation of GSH in response to an LPS-mediated increase in nitric oxide production. Thus, astrocytes respond to oxidative stress by increasing their reducing power via the stimulation of the pentose phosphate pathway. Steele and co-workers investigated the response of cultured human U373 astrocytic cells to the incubation of the cells for up to 96 h with increasing concentrations of the pro-inflammatory cytokines IL-1β and TNFα [65]. Whilst both the intracellular and extracellular concentration of GSH increased, the extracellular concentration of cysteinylglycine fell significantly. Based upon these results, the authors speculated that the chronic activation of astrocytes could render the cells unable to supply sufficient quantities of GSH to neurons. The mechanism behind cytokine-mediated changes in astrocytic GSH production is unclear, particularly in regard to the source of cysteine. Jackman et al. [66] note that the expression of xCT in astrocytes (but not neurons or oligodendrocytes) is increased in response to IL-1β in vitro. A more recent study by the same group [67] shows that IL-1β promotes a stabilisation of xCT-specific mRNA in primary astrocyte cultures that increases its half-life. Interestingly, the authors’ data shows an increase in GSH production that is absent in cells lacking xCT. Furthermore, the IL-1β-mediated increase in GSH was sufficient to protect astrocytes from oxidative damage [68]. These authors also showed that IL-1β activation involves a translocation of the RNA-binding protein, HuR, from the nucleus to the cytoplasm [67]. They conclude that further investigations into the regulation of xCT should acknowledge the critical role played by this protein in the regulation of xCT expression and activity.

Less is known concerning the impact of astrocyte activation on the TS pathway, particularly in regard to the provision of GSH. The expression of CBS increases in activated astrocytes following kainate-induced seizures in the hippocampus [56]. In contrast, the pro-inflammatory cytokines TNFα and IL-16 decrease the expression of CBS in astrocytes; this has been linked to a decrease in H_2_S production, and is in line with the anti-inflammatory properties of the gas [53]. Data on the response of CTH to astrocyte activation is scarce, although results from the author’s laboratory indicate that the enzyme is upregulated in response to cytokine activation in primary astrocytes [51], and signals that CBS and CTH may be independently regulated. In a study of THP-1 macrophages, it is reported that miRNA-186 binds to the 3’UTR of CTH, causing inhibition and a consequent increase in pro-inflammatory cytokine secretion [69]. Note that, in this case, a change in the activity of CTH precedes cytokine secretion, rather than follows it.

Many of the cytokine-mediated responses in astrocytes may be mediated by the activation of Nrf2. For example, Nrf2 is activated by TNFα, and mediates the observed increase in GSH in human U373 astrocytes via an increased expression of GCL [65]. Interestingly, persistent low-level stimulation of astrocytic N-methyl-D-aspartate (NMDA) receptors stabilises Nrf2 by a Cdk5-mediated pathway [33]. This causes an increase in GSH production via promoting the expression of antioxidant-related genes that include xCT and GCL. The γ-glutamyltransferase inhibitor, acivicin, prevented the astrocytic NMDA-mediated increase in supply of GSH precursors to neurons, thus confirming the dependence of neurons on astrocytes for the provision of GSH for neuronal antioxidant purposes. These results support the discussion presented earlier, that the upregulation of astrocyte GSH meets demands for export, and does not occur for in situ antioxidant purposes. However, there is much still to be learned about the possibilities of increasing Nrf2 responses to oxidative stress in astrocytes, particularly in relation to the prevention of disruptions to thiol redox homeostasis in neurodegenerative disease.

## 6. Cysteamine: A Potential Therapeutic?

Cysteamine, and its oxidised form, cystamine, have been proposed as potential neuroprotective agents that may have benefit in the treatment of neurodegenerative disease [70]. However, cysteamine is a strong oxidant that, in the presence of transition metals, generates several pro-oxidant intermediates, including H_2_O_2_, superoxide, and hydroxyl and thiyl radicals. During the 1990s, Shipper and colleagues published several papers on the effects of cysteamine treatment on astrocytic metabolism and well-being [71,72,73]. The common theme was the formation of cellular (cytosolic) inclusions arising from the treatment of astrocytes with cysteamine, which were identified as peroxide-positive granules. It was concluded that cysteamine-related disruptions to astrocyte antioxidant defences may contribute to both oxidative mitochondrial injury and the development of enhanced cytoprotection, which was likely linked to the upregulation of hemeoxygenase-1. These investigators have reported that cysteamine produces astrocytic hypertrophy, in other words, astrocyte activation [74]. The long-term administration of cysteamine to animals produces locomotor defects, as illustrated by poor performance in the Morris Water Maze compared to normal controls. Morphologically, chronic cysteamine treatment produces a senescent phenotype in astrocytes (i.e., iron-laden mitochondria) that accumulate in several regions, including the hippocampus and striatum [74]. Nevertheless, chronic cysteamine treatment produces some positive outcomes, as indicated by its ability to reverse motor deficits in the 6-hydroxydopamine model of Parkinson’s disease [75]. Indeed, these beneficial effects have prompted calls for the exploration of cysteamine in clinical trials in Parkinson’s disease patients. Cysteamine is used in the treatment of the rare lysosomal storage disease, cystinosis, which is characterised by the dysfunction of a lysosomal cystine transporter that results in the accumulation of cystine crystals in cells and tissues [76]. A disulfide exchange of cysteamine with cystine forms cysteine and alleviates the symptoms of the disease. To date, little research has been performed on the impact of long-term cysteamine treatment on cerebral thiol redox homeostasis. On the down-side, cysteamine has a high rate of metabolism that, coupled with the unpalatability of orally-administered cysteamine, questions its usefulness in the wider treatment of neurodegenerative disorders.

## 7. Conclusions

Many questions remain regarding the relationship between the *x_c_^−^*-exchanger and the TS pathway in the provision of cysteine for GSH in astrocytes. The fact that the intracellular concentration of cysteine is typically 100-fold lower than that of GSH, and that the amino acid also serves as a precursor of H_2_S, implies that the availability of cysteine must be under strict regulation. The necessity of maintaining GSH under conditions of oxidative stress or astrocyte activation demands a level of flexibility and “cross-talk” between these complementary systems that is not yet fully understood. The role of cystathionine in contributing to thiol redox homeostasis in astrocytes requires further investigation.
